# Prevalence and patterns of psychiatric comorbidities in type 2 diabetes mellitus: a systematic review and meta-analysis

**DOI:** 10.3389/fpubh.2026.1697079

**Published:** 2026-02-17

**Authors:** Muhanad Saleh Alzahrani, Abdullah Alabbasi, Ziyad Alzahrani, Turki Alsafrani

**Affiliations:** 1 Jeddah First Cluster, Joint Program of Family Medicine Jeddah, Jeddah, Saudi Arabia; 2 College of Medicine, King Saud bin Abdulaziz University for Health Sciences, Jeddah, Saudi Arabia; 3 King Abdullah International Medical Research Center, Jeddah, Saudi Arabia; 4 Department of Orthopedic Surgery, Saudi German Hospital Jeddah, Jeddah, Saudi Arabia

**Keywords:** cognitive impairment, depression, meta-analysis, prevalence, psychiatric comorbidities, schizophrenia, type 2 diabetes mellitus

## Abstract

**Background:**

Type 2 diabetes mellitus (T2DM) is a global health challenge that is frequently complicated by psychiatric comorbidities, which can worsen disease outcomes and quality of life. Accurate prevalence estimates are essential to inform integrated management strategies.

**Objective:**

This study aimed to systematically review and meta-analyze the prevalence and patterns of psychiatric comorbidities in individuals with T2DM.

**Methods:**

A comprehensive search of PubMed, ScienceDirect, Google Scholar, and the Cochrane Library was conducted according to the Preferred Reporting Items for Systematic Reviews and Meta-Analyses (PRISMA) guidelines. Eligible studies included full-text, peer-reviewed articles reporting psychiatric comorbidities in adults with T2DM. Data extraction was performed independently by two reviewers. Pooled prevalence estimates were calculated using a random effects model, with heterogeneity assessed using the I^2^ statistic. The search was updated through July 2025.

**Results:**

A total of 12 studies met the inclusion criteria. The pooled prevalence of depression in adults with T2DM was 19% (95% CI: 14–24%), based on eight contributing studies. Mild cognitive impairment (MCI) was also frequently observed, with a pooled prevalence of 39% (95% CI: 23–59%) across four studies. Considerable variability among studies was evident, likely attributable to differences in study design, diagnostic methods, sampling frames, and population characteristics.

**Conclusion:**

Psychiatric comorbidities are highly prevalent in individuals with T2DM, particularly depression and cognitive impairment, emphasizing the need for integrated psychiatric and metabolic care. This meta-analysis demonstrates that psychiatric comorbidities, particularly depression and cognitive impairment, are common in individuals with T2DM, highlighting the need for routine psychiatric screening and integrated diabetes care.

## 1 Introduction

Psychiatric disorders are highly prevalent and are major contributors to global disability and disease burden (1). Individuals with severe mental illness experience a reduced life expectancy of approximately 15–20 years, largely attributable to cardiometabolic disease and preventable physical health conditions (2, 3). Beyond factors such as suicide, accidents, and high-risk behaviors, heart disease is a major contributor to the premature mortality observed in this population (4, 5). Rates of cardiovascular disease are significantly higher among individuals with psychiatric disorders compared to the general population, contributing to excess premature mortality. Among the diverse risk factors associated with heart disease, metabolic syndrome warrants particular attention. Type 2 diabetes mellitus (T2DM) serves as an important component (6). Globally, T2DM is a pressing public health challenge. In 2021, it was estimated that 537 million people were living with T2DM, with projections indicating a 46% increase to 783 million by 2045 (7). A recent meta-analysis demonstrated a connection between depression and hyperglycemia in individuals with diabetes (8), as well as an increased risk of diabetes-related complications (9). In addition, evidence from three controlled trials suggests that effective treatment of depression can improve glycemic control (10–12). Therefore, establishing an accurate estimate of the prevalence of depression among patients with type 2 diabetes is necessary to better understand the potential effects of managing depression in this population. Evidence suggests that individuals with psychiatric disorders exhibit greater disturbances in glucose regulation and insulin sensitivity than healthy counterparts (13–16). Multiple mechanisms may explain this association. From a genetic perspective, psychiatric disorders and T2DM share partially overlapping genetic risk architecture (17). For example, the transcription factor gene TCF7L2, one of the strongest known genetic risk loci for T2DM (18), has also been implicated in susceptibility to psychiatric disorders (19). Lifestyle factors, including sedentary behavior and poor dietary patterns, further contribute to elevated metabolic risk in psychiatric populations (20).

### 1.1 Rationale

T2DM is a chronic metabolic disorder that poses a significant global health burden due to its high prevalence, complications, and related mortality. Terminology for type 2 diabetes follows contemporary nomenclature consistent with the Diabetes Care 2025 Supplement recommendations. Beyond its physical health results, there is increasing recognition of psychiatric comorbidities in individuals with T2DM, including depression, anxiety disorders, schizophrenia, and cognitive impairment. These conditions not only increase the burden of the disease but also affect glycemic control, adherence to treatment, and life, leading to worse clinical outcomes. While previous studies have examined individual psychiatric conditions in terms of T2DM, reported prevalence rates vary widely due to differences in study design, population characteristics, and clinical approaches. A comprehensive synthesis of the available evidence is therefore required to better understand the distribution of psychiatric comorbidities in T2DM and their implications for patient care.

### 1.2 Objectives

This review aimed to synthesize existing evidence on the prevalence and patterns of psychiatric comorbidities including depression, anxiety, cognitive impairment, and schizophrenia among adults with T2DM.

## 2 Methodology

### 2.1 Study design

This review is a systematic assessment and meta-analysis of scientific studies, guided by the Preferred Reporting Items for Systematic Reviews and Meta-Analyses (PRISMA) (21) reporting guidelines. This systematic review and meta-analysis (SRMA) has been registered with PROSPERO (22), the International Prospective Register of Systematic Reviews, ensuring transparency and adherence to the PRISMA review protocols.

### 2.2 Inclusion criteria

The inclusion criteria were as follows:

1. Studies including adult patients (≥18 years) with T2DM.
2. Studies reporting psychiatric comorbidities or patterns of psychiatric conditions, not limited to depression, anxiety, schizophrenia/psychological disorders, and cognitive loss.
3. Observational studies (cross-sectional, case–control), randomized controlled trials (RCTs), and baseline data from clinical studies that report the prevalence of psychiatric comorbidities.
4. Studies providing quantitative data (e.g., prevalence rates, ratios, or adequate raw data) to calculate the prevalence of psychiatric conditions in T2DM.
5. Articles published in English.

### 2.3 Exclusion criteria

The exclusion criteria included the following:

1. Studies involving type 1 diabetes, gestational diabetes, or mixed populations without separate reporting for T2DM.
2. Case reports, case series, reviews, commentaries, editorials, and conference abstracts that do not provide adequate data.
3. Studies in which psychiatric comorbidity data are not clearly reported or cannot be extracted.
4. Studies involving pediatric populations (<18 years).
5. Animal studies or studies that do not include human participants.

### 2.4 Search strategy

The literature search covered protected databases along with PubMed, Google Scholar, and the Cochrane Library. For Google Scholar, the first 300 records sorted by relevance were screened, following standard practice to manage the output volume. Duplicates were removed manually, and only peer-reviewed full-text articles were considered. The PRISMA guidelines were followed throughout the search process. Boolean operators (AND/OR) were applied to refine the search terms and ensure specificity. The search strategy used was: (“Diabetes Mellitus, Type 2”[Mesh] OR “type 2 diabetes”[tiab] OR “T2DM”[tiab])

AND

(“Mental Disorders”[Mesh] OR “psychiatric disorder*”[tiab] OR “psychiatric comorbidit*”[tiab] OR “psychological disorder*”[tiab]

OR “depression”[Mesh] OR depression[tiab] OR depressive[tiab]

OR “anxiety”[Mesh] OR anxiety[tiab]

OR “schizophrenia”[Mesh] OR schizophrenia[tiab] OR psychosis[tiab]

OR “cognitive dysfunction”[Mesh] OR “cognitive impairment”[tiab] OR dementia[tiab])

AND

(“Prevalence”[Mesh] OR prevalence[tiab] OR epidemiology[tiab] OR frequency[tiab] OR burden[tiab] OR “risk factors”[tiab]).

The search was initially conducted in 2023 and was last updated on 15 January 2025 to ensure inclusion of the most recent evidence.

### 2.5 Data collection

A total of two reviewers independently investigated all records identified through the database search. Initial screening was performed at the title and abstract levels to exclude irrelevant studies, including those not focused on type 2 diabetes mellitus (T2DM), those that did not report psychiatric comorbidities, or those that were reviews, case reports, or conference abstracts lacking adequate data. Potentially eligible studies were evaluated in detail against predetermined inclusion and exclusion criteria. Any disagreements between reviewers were resolved through discussion, and a third reviewer was consulted when consensus could not be reached.

### 2.6 Data extraction

Data extraction was performed independently by two reviewers using a predesigned standardized form. Extracted information included study characteristics (first author, publication year, country, and study design), sample size, and population characteristics (mean age, sex distribution, and diabetes duration). Psychiatric comorbidities assessed included depression, anxiety, schizophrenia, bipolar disorder, and cognitive impairment, along with the diagnostic tools or criteria used. Outcome measures included the prevalence of psychiatric comorbidities, patterns of distribution (e.g., type, severity, and regional variations), and any reported associations with demographic or clinical variables such as glycemic control, diabetes complications, or treatment adherence. Data on race/ethnicity, socioeconomic status, urban–rural residence, and medication use were extracted when available. However, these variables were inconsistently reported across studies and, therefore, were not included in pooled quantitative analyses. Medication exposure was documented descriptively, given its potential influence on metabolic and psychiatric outcomes.

### 2.7 Data synthesis

Pooled prevalence estimates of psychiatric comorbidities in T2DM were calculated using a random effects model to account for heterogeneity among studies. For each study, prevalence rates were extracted as the number of events (patients with a psychiatric disorder) and the total sample size. Subgroup analyses were conducted based on the type of psychiatric comorbidity, geographic region, study design, and diagnostic method. Statistical heterogeneity was assessed using Cochran’s Q test and quantified with the I^2^ statistic. Sensitivity analyses were performed by excluding studies with a high risk of bias or small sample sizes to evaluate the robustness of the results. Potential publication bias was examined through visual inspection of funnel plots and Egger’s regression test.

### 2.8 Quality assessment of trials

A total of two authors independently evaluated the quality of the articles using the Newcastle–Ottawa scale (NOS) tool. Any disagreements were resolved by consensus.

### 2.9 Statistical analysis

Statistical analyses were performed using R Studio. A two-sided *p*-value of <0.05 was considered statistically significant.

## 3 Results

### 3.1 Study items

**Figure 1:**
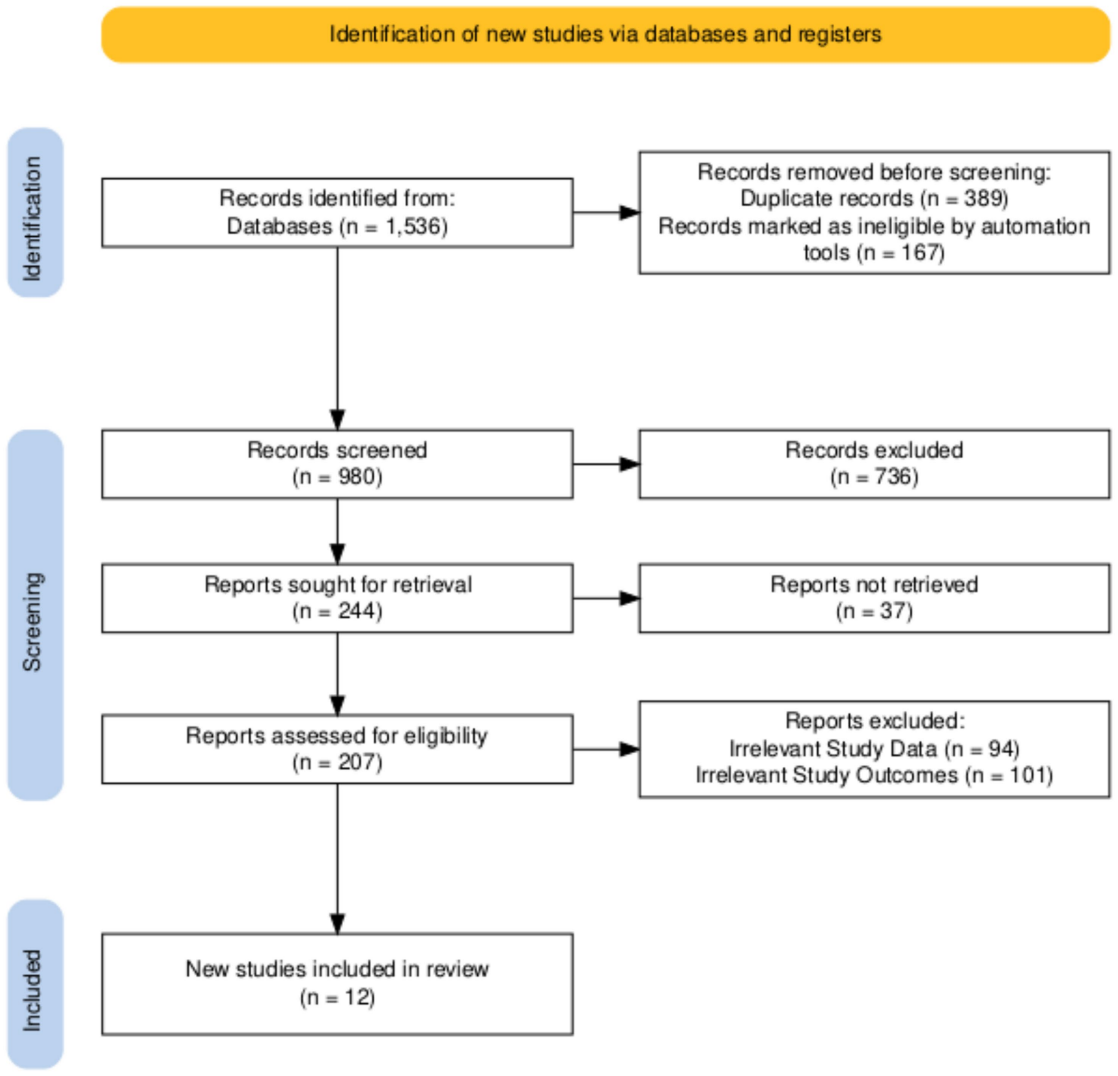
PRISMA flowchart of the included studies.

A PRISMA flowchart was created for the included studies. The PRISMA flowchart is a widely used tool to illustrate the study selection process in systematic reviews and meta-analyses. By documenting the number of studies at each step and the reasons for exclusions, the PRISMA flowchart provides readers a clear understanding of the methodology and rigor underlying the study selection process (23). The flowchart is presented in Figure 1.

### 3.2 Study characteristics

Some included studies (e.g., intervention or incidence-focused cohorts) did not contribute to pooled prevalence estimates but were retained because they provided relevant baseline psychiatric data or contextual information. Of the 908 records screened, 736 were excluded due to irrelevant populations, absence of psychiatric outcome reporting, insufficient extractable prevalence data, duplicate publications, or non-original study designs (e.g., reviews, editorials, or conference abstracts).

The characteristics of all included studies are provided in Table 1.

**Table 1:** Characteristics of the included studies.

Author (year)	Study design	Country	Sample size	Prevalence	Follow-up (years)	Age (mean/median)	T2DM diagnosis criteria	Psychological comorbidity (specific conditions)	Depression assessment method
Shamsutdinova et al. (2023) (26)	Cross-sectional sample	UK (East London)	10,159 individuals with SMI (analytical sample, from 674,000 adults).	T2DM prevalence in the SMI sample: 14.9% (*N* = 1,513 cases). The overall T2DM rate was 2.7 times higher in individuals with SMI vs. the general population (14.9% vs. 5.6%).	NA (cross-sectional prevalence model)	Mean age of 44.4 years (SD ± 13.2) in the SMI sample.	Past diagnosis based on diagnostic codes and pharmacy records.	Severe mental illness (SMI), an umbrella term including schizophrenia, bipolar disorder, and other non-organic psychoses; depression.	Past diagnoses of depression were established using relevant clinical codes.
Abbas et al. (2023) (34)	Randomized control trial (RCT), prospective	Pakistan (Faisalabad)	90 diagnosed T2DM patients (45 experimental, 45 waitlist control) from 130 initially screened. 62 patients completed procedures.	This study investigates the efficacy of CBT, not T2DM prevalence. Global T2DM prevalence is 422 million, projected to rise to 548 million in 25 years. T2DM prevalence in urban areas of Pakistan: 13.7%. Global Diabetes Distress (DD) prevalence: ~45%.	16 weeks (pre- and post-assessment stages)	Mean age of 36.93 years (SD 6.87) for the experimental group and 37.62 years (SD 6.77) for the control group. Age range: 23 to 50 years.	Diagnosis confirmed by medical consultants using medical reports.	Diabetes distress (DD), depressive symptoms, and health anxiety.	The Patient Health Questionnaire (PHQ) for depressive symptoms; the Diabetes Distress Scale (DDS) for diabetes distress.
Dalsgaard et al. (2024) (28)	Cross-sectional, population-based survey	Denmark (Central Denmark Region)	18,222 respondents with T2DM (from 39,766 invited; 46% response rate)	Prevalence of depression only: 11%. Prevalence of diabetes distress only: 14%. Prevalence of both: 4%. 71% of individuals had neither condition.	NA (cross-sectional)	Median age of 66 years (IQR: 58; 72 years) for responders. Age by group: Neither: 67 (60; 72); Depression only: 64 (57; 71); Diabetes distress only: 62 (55; 69); Both: 59 (52; 66).	Identified through an algorithm utilizing national Danish registers.	Depression and diabetes distress.	Register-based method: Hospital admission for depression in the last 2 years or at least two antidepressant prescriptions in the year prior to the survey. Risk of depression was assessed using the WHO-5 Well-Being Index.
Kanwar et al. (2019) (35)	Descriptive cross-sectional study	India (North India, Shimla)	202 eligible T2DM patients enrolled (from 320 screened)	Overall psychiatric comorbidity: 58.4%. Depression: 41.9%. Dysthymia: 9.4%. Generalized anxiety disorder: 6.8%. Panic disorder: 6.0%. Social phobia: 2.6%. Agoraphobia: 0.9%.	NA (cross-sectional)	Mean age of 50.63 ± 09.37 years. Age range: 24–60 years.	Diagnosis made by medical department.	Depression, dysthymia, generalized anxiety disorder, panic disorder, social phobia, and agoraphobia.	Hamilton Depression Rating Scale (HAM-D) for severity. Mini-International Neuropsychiatric Interview 6.0 (M. I. N. I.6.0) for screening major Axis I disorders.
Yang et al. (2020) (24)	Longitudinal observational study	China (Beijing)	157,570 adult psychiatric inpatients	Overall T2DM prevalence: 10.75% (95% CI 10.60 to 10.90%). T2DM prevalence among schizophrenia patients: 11.63%. T2DM prevalence in other psychiatric disorders: 10.17%. Annual T2DM prevalence increased from 5.20% in 2005 to 12.71% in 2018.	2005 to 2018 (14 years)	Mean age of 43.53 ± 17.16 years	ICD-10 codes: E11 (non-insulin-dependent DM) and E14 (unspecified DM).	Schizophrenia, bipolar affective disorder, recurrent depressive disorder, depressive episode, mental and behavioral disorders due to the use of alcohol, acute and transient psychotic disorders, persistent delusional disorders, manic episode, obsessive-compulsive disorder, and schizoaffective disorders; antipsychotic medication use.	ICD-10 codes for depressive disorder, recurrent depressive disorder, and depressive episode.
Lambert et al. (2023) (29)	Observational study (tertiary referral clinics)	Australia (Sydney)	1,402 individuals with SMI	Overall diabetes prevalence: 23.0% (*n* = 322). Pre-diabetes: 19.5%. Newly diagnosed diabetes: 15.8% of those with diabetes.	NA (data collected over 5 years, 2014–2019, for baseline visit)	Mean age of 43.9 ± 12.8 years.	Hierarchical approach: HbA1c ≥ 6.5%, fasting blood glucose (FPG) ≥ 7.0 mmol/L, or self-reported diagnosis with prescription of antihyperglycemic medication.	Severe mental illness (SMI) including schizophrenia (63.5%), schizoaffective disorder, bipolar disorder, depression, and other psychoses.	Depression was diagnosed using ICD-10 codes.
Jackson et al. (2019) (36)	Retrospective cohort study, national population-based	Scotland, U.K.	254,136 diabetes cases identified during 2001–2015. Incident cases: 246,046 (no mental illness), 2,315 (schizophrenia), 1,720 (bipolar disorder), 4,055 (depression).	The study focuses on the incidence of T2DM. Incidence rates in 2015 were 1.5–2.5 times higher in individuals with vs. without a major mental disorder, with the gap slightly increasing over time.	2001–2015 (15 years)	Average age at T2DM onset: Schizophrenia: 51.4 ± 12.6 years; Depression: 57.6 ± 13.1 years; Bipolar disorder: 60.0 ± 12.1 years; No mental illness: 60.8 ± 13.2 years.	Scottish Care Information–Diabetes (SCI-Diabetes) dataset, algorithm based on clinician diagnosis, prescription data, and age at diagnosis.	Schizophrenia, bipolar disorder, and depression.	Depression was diagnosed using hospital records with ICD-10 codes (F32-F34).
Gao et al. (2015) (25)	Cross-sectional study, cluster random sampling	China (Tianjin)	8,213 individuals aged 65+; 1,109 participants with T2DM (screened). Among T2DM patients, 690 had MCI, 132 had dementia, and 287 were cognitively intact.	Overall MCI with T2DM prevalence: 13.5% (among all participants). Dementia with T2DM prevalence: 2.34% (among all participants). Among T2DM patients, MCI was 62.2% (*n* = 690) and dementia was 11.9% (*n* = 132).	NA (cross-sectional)	Mean age of 72.4 years (±3.5) for enrolled participants. T2DM-MCI: 74.3 ± 3.5 years; T2DM-Demented: 77.4 ± 4.5 years; T2DM-Cognitively intact: 70.9 ± 4.7 years.	WHO recommendations modified by American Diabetes Association criteria (1997) (insulin/oral hypoglycemic agents, FBG > =7.0 mmol/L, or 2-h OGTT > = 11.1 mmol/L).	Mild cognitive impairment (MCI), dementia. Depression (noted in medical history but not as primary comorbidity prevalence).	Medical history collection.
Huang et al. (2019) (37)	Cross-sectional study	China (Nanjing)	352 T2DM patients (208 male individuals, 144 female individuals); 157 T2DM patients had MCI, and 195 had normal cognition.	Prevalence of MCI in T2DM patients: 44.6% (157/352)	NA (cross-sectional)	MCI patients were older than normal controls. Age range: 40–80 years.	WHO criteria (1999) (FBG > = 7 mmol/L, random blood glucose > = 11.1 mmol/L, or 2 h blood glucose > = 11.10 mmol/L after 75 g OGTT; asymptomatic patients require additional blood glucose confirmation). History of diabetes >3 years.	Mild cognitive impairment (MCI). Hypertension, hyperuricemia, coronary artery disease, diabetic nephropathy, and diabetic retinopathy.	A self-rating depression scale was included as part of neuropsychological tests (but it was not used for prevalence outcomes).
Lee et al. (2023) (33)	Large-scale prospective cohort study	South Korea	6,457,991 young adults (20–39 years). 658,430 individuals with psychiatric disorders.	The study focuses on the incidence of T2DM. Incidence rates of T2D for individuals with and without psychiatric disorders were 2.89 and 2.56 per 1,000 person-years, respectively. Incidence rates of T2DM per 1,000 person-years: Schizophrenia, 6.05; Bipolar disorder, 5.02; Depressive disorder, 3.00; Anxiety disorder, 2.78; and Sleep disorder, 3.23.	Mean 7.59 years (up to Dec 2018)	Mean age of 30.74 years (SD 4.98). Age groups: 20–29 years and 30–39 years.	T2D: At least one service claim of ICD-10-CM codes E11, E12, E13, or E14, and a prescription for at least one antidiabetic drug. In addition, FPG > = 126 mg/dL without an antidiabetic medication claim.	Schizophrenia (F20), bipolar disorder (F30-F31), depressive disorder (F32-F33), anxiety disorder (F40-F41), and sleep disorder (G47 and F51).	ICD-10-CM codes F32-F33 for depressive disorder.
Li et al. (2019) (27)	Cross-sectional study, cluster random sampling	China (national study, various communities)	3,246 people aged 60 + screened. 256 participants diagnosed with T2DM. Among T2DM patients, 56 had MCI.	Prevalence of MCI in T2DM patients: 21.8% (56/256).	NA (cross-sectional)	Mean age for T2DM-MCI: 74.23 ± 6.93 years. T2DM-Normal: 71.12 ± 7.51 years. No-T2DM-MCI: 75.90 ± 7.57 years.	American Diabetes Association criteria (self-reported physician diagnosis or treatment with insulin/oral hypoglycemic agents, FBG > =7.0 mmol/L, or 2-h OGTT > = 11.1 mmol/L).	Mild cognitive impairment (MCI), depression.	The Patient Health Questionnaire (PHQ-9) and Geriatric Depression Scale (GDS) were used to screen and exclude depression.
Xia et al. (2020) (30)	Cross-sectional analyses	China (Wuhan)	297 T2DM patients enrolled (from 591 screened). 47 in the dementia group, 174 in the MCI group, and 76 with normal cognition.	Prevalence of dementia in T2DM patients: 15.8% (*n* = 47). Prevalence of MCI in T2DM patients: 58.6% (*n* = 174). The cognitive dysfunction rate (MCI + dementia) was nearly twice as high as in healthy people.	NA (cross-sectional)	Average age of 56.8 years (SD 6.9). Age range: 45–74 years. MCI group mean age: 57.1 years; Normal cognition group mean age: 55.0 years.	American Diabetes Association (ADA) diagnostic criteria (FPG > =7.0 mmol/L and/or PPG > =11.1 mmol/L, random plasma glucose > = 11.1 mmol/L with symptoms, or HbA1c > =6.5%).	Mild cognitive impairment (MCI), dementia. Depression, anxiety, and delirium (exclusion criteria).	Mental and neurological disorders such as depression, anxiety, and delirium were used as exclusion criteria.

### 3.3 Meta-analysis

#### 3.3.1 Prevalence of depression

**Figure 2:**
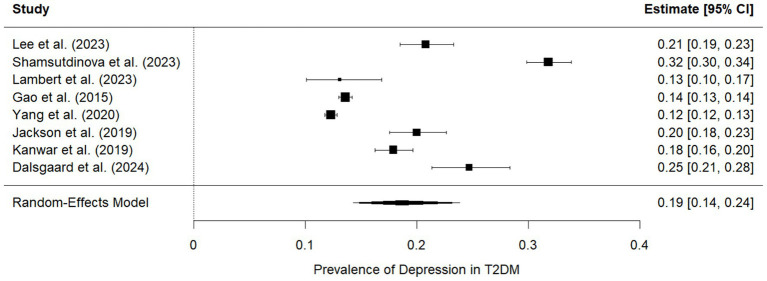
Forest plot showing the prevalence of depression among adults with T2DM.

A pooled analysis of eight studies evaluating the prevalence of depression among individuals with T2DM demonstrated substantial variability, with prevalence estimates ranging from 12 to 32%. The lowest prevalence was reported by Yang et al. (24) (12%; 95% CI: 0.12–0.13) and Gao et al. (25) (14%; 95% CI: 0.13–0.14), whereas the highest prevalence was observed by Shamsutdinova et al. (26) (32%; 95% CI: 0.30–0.34). Despite this heterogeneity, the random effects model yielded a pooled prevalence of 19% (95% CI: 14–24%), indicating that approximately one in five adults with T2DM experience clinically significant depressive symptoms. The variation in individual study estimates likely reflects differences in study populations, diagnostic criteria, and methodological characteristics. Nonetheless, the overall summary effect supports the conclusion that depression represents a common psychiatric comorbidity in T2DM (Figure 2).

#### 3.3.2 Prevalence of mild cognitive impairment

A total of four studies reported the prevalence of mild cognitive impairment (MCI) among individuals with T2DM. Considerable between-study variation was observed, with prevalence estimates ranging from 22 to 62%. Li et al. (27) reported the lowest prevalence at 22% (95% CI: 0.17–0.27), whereas Gao et al. (25) reported the highest prevalence at 62% (95% CI: 0.59–0.65). Using a random effects model, the overall pooled prevalence of MCI in T2DM was 39% (95% CI: 23–59%), indicating that cognitive impairment is a common and clinically relevant comorbidity within this population. The wide confidence interval surrounding the pooled estimate reflects heterogeneity in diagnostic assessments, cognitive testing tools, population characteristics, and the thresholds used to define impairment across studies. Despite this heterogeneity, the findings indicate that early cognitive decline is highly prevalent in T2DM and may warrant routine screening in clinical practice.

#### 3.3.3 Prevalence of schizophrenia in T2DM

**Figure 3:**
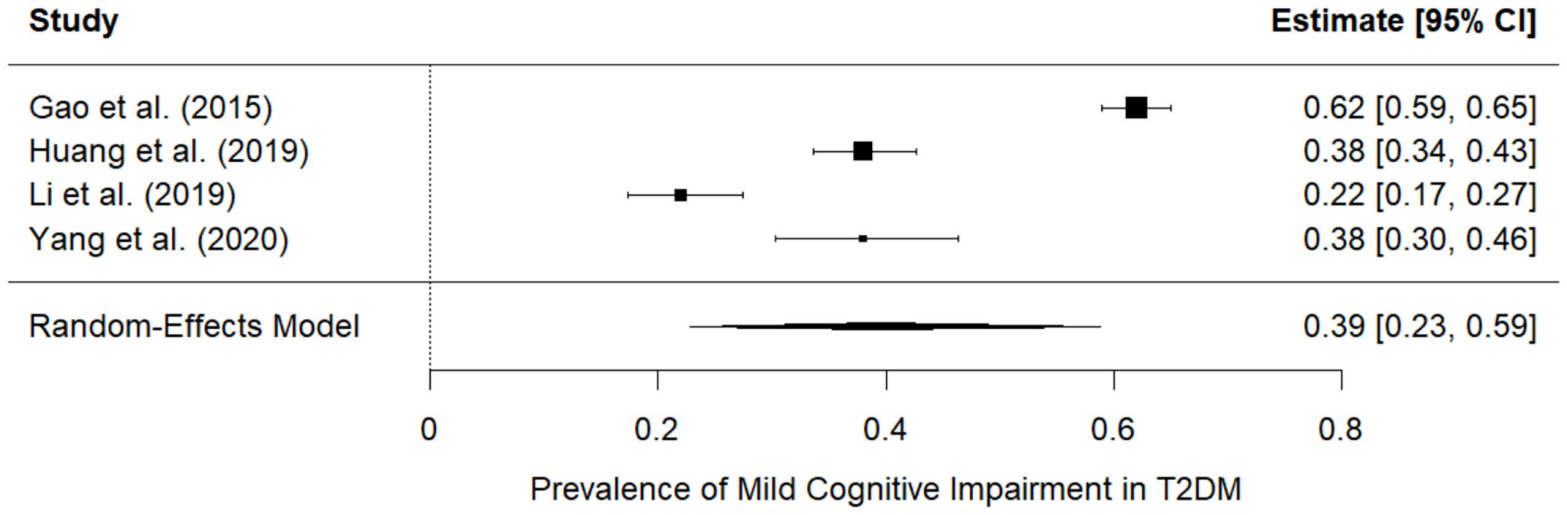
Prevalence of MCI in T2DM.

Only two studies reported schizophrenia prevalence in T2DM, with highly divergent estimates driven by differences in population settings (psychiatric inpatients vs. the general population). Due to extreme heterogeneity (I^2^ = 100%), a pooled estimate was not calculated (Figure 3).

### 3.4 Publication bias

Publication bias among the included studies is presented below.

**Figure 4:**
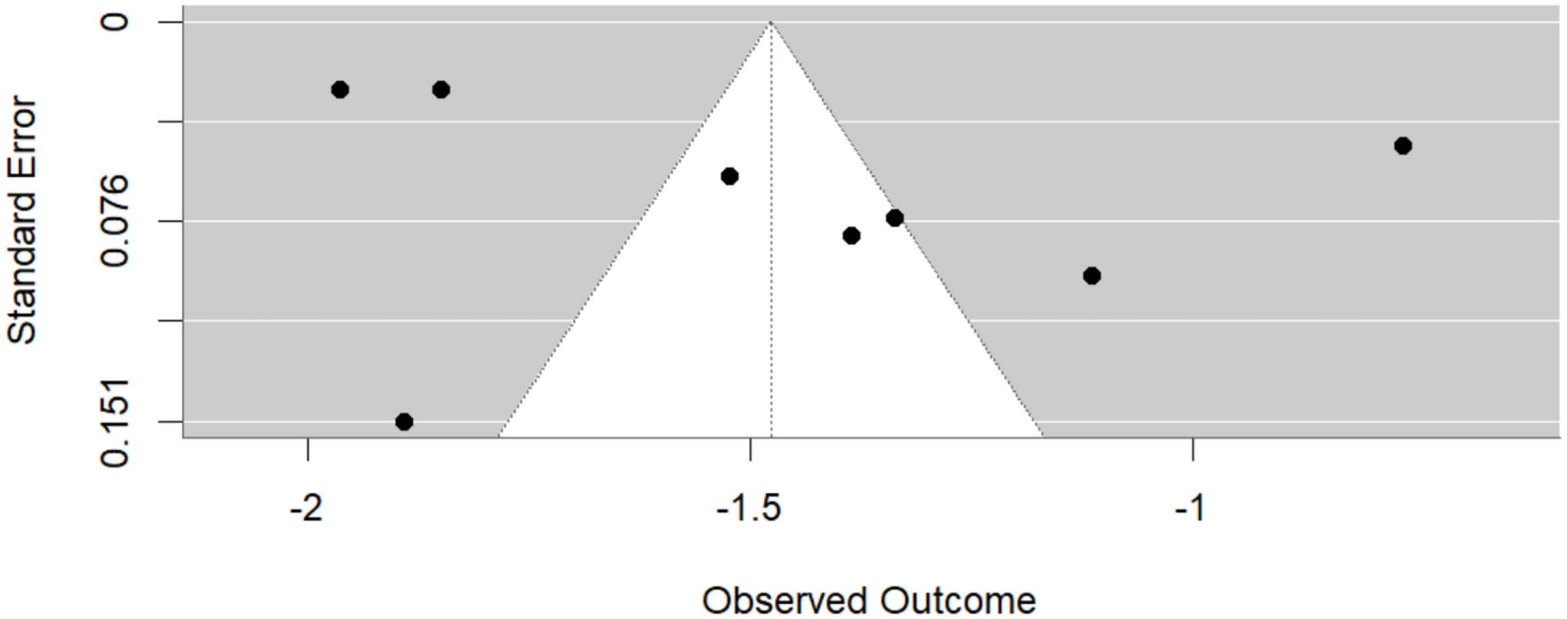
Funnel plot showing publication bias.

The quality of the included studies was assessed using the Newcastle–Ottawa scale (NOS), which assigns stars based on three domains: Selection (0–4 stars), Comparability (0–2 stars), and Outcome (0–3 stars), for a maximum total of 9 stars (Figure 4; Table 2).

**Table 2:** Quality assessment.

Study	Selection (0–4)	Comparability (0–2)	Outcome (0–3)	Total stars	Quality level
Shamsutdinova et al. (2023) (26)	★★★★	★	★★	7	High
Abbas et al. (2023) (34)	★★★	★	★★	6	Moderate
Dalsgaard et al. (2024) (28)	★★★	★	★★	6	Moderate
Kanwar et al. (2019) (35)	★★★★	★★	★★	8	High
Yang et al. (2020) (24)	★★★	★	★★	6	Moderate
Lambert et al. (2023) (29)	★★★	★	★★	6	Moderate
Jackson et al. (2019) (36)	★★★★	★★	★★★	9	High
Gao et al. (2015) (25)	★★★	★	★★	6	Moderate
Huang et al. (2019) (37)	★★★	★	★★	6	Moderate
Lee et al. (2023) (33)	★★★★	★★	★★	8	High
Li et al. (2019) (27)	★★★	★	★★	6	Moderate
Xia et al. (2020) (30)	★★★	★	★★	6	Moderate

## 4 Discussion

Our meta-analysis shows that psychiatric comorbidities are an important concern in adults with T2DM, although the magnitude varies substantially by condition. A total of eight studies contributed prevalence data for depression, yielding a pooled prevalence of 19% (95% CI: 14–24%), indicating that approximately one in five adults with T2DM experiences clinically relevant depressive symptoms. Cognitive impairment—primarily mild cognitive impairment (MCI)—was also common. Based on four eligible studies, the pooled prevalence of MCI was 39% (95% CI: 23–59%), reflecting substantial between-study variability while confirming that early cognitive decline is a common comorbidity in T2DM. In contrast, schizophrenia appeared uncommon in this population. For schizophrenia, the available evidence was insufficient to conduct a meta-analysis. Only two studies reported prevalence, and their findings were highly divergent due to differences in population settings. Since heterogeneity was extreme (I^2^ = 100%), a pooled estimate was not calculated, and the results were interpreted narratively. No meta-analysis could be performed for anxiety or bipolar disorder due to insufficient data. Overall, our findings indicate that depression and cognitive impairment are prevalent and clinically significant comorbidities in T2DM, whereas schizophrenia remains relatively rare, and the limited evidence precludes firm conclusions for other psychiatric disorders. For example, Dalsgaard et al. reported that only approximately 11% of Danish T2DM patients met registry-defined criteria for depression (28), and Kanwar et al. reported a 41.9% depression rate in Indian T2DM outpatients (29). These individual prevalence figures are substantially lower than our pooled estimate, suggesting that our meta-analysis may reflect differences in study populations or methods (see below). Conversely, our pooled MCI prevalence rate (39%) is broadly consistent with the highest reported rates. Gao et al. found MCI in 62.2% of older Chinese T2DM patients (25), and Xia et al. reported 58.6% in a Chinese cohort (30), both similar to our pooled result. Other studies reported lower MCI prevalence [e.g., 44.6% (27) and 21.8% (25)], indicating variability. For schizophrenia, the two included studies produced contradictory results: Lambert et al. (using a tertiary clinic sample of severe mental illness) found that 73% of patients with schizophrenia had comorbid diabetes (25) (a very high rate), whereas Lee et al. reported much lower schizophrenia prevalence in a large young adult cohort, reflecting differences in sampling frame, age distribution, and clinical characteristics. In summary, our findings align with some individual studies (especially those reporting high estimates of depression and MCI) but differ markedly from others, highlighting discrepancies across study settings and methods. Other meta-analyses reported similar results (31, 32).

Possible sources of heterogeneity include geographic and population differences (the studies spanned Europe, Asia, and Australia), as well as variations in diagnostic approaches. For example, Dalsgaard et al. used national registry data (ICD codes and prescription records) to identify depression (28), whereas Kanwar et al. used structured clinical interviews and rating scales (29). Cognitive impairment studies involved older adults from China and used different screening tools (27, 33), resulting in variations in age and study setting. The schizophrenia findings were influenced by one clinic-based SMI sample (25) versus a general population cohort (30), which differed greatly in age (mean ~44 vs. 30 years) and clinical context. Diagnostic criteria also varied: Depression was assessed using the WHO-5 Well-Being Index in one study (28) and the Hamilton Depression Rating Scale in another (29); T2DM was defined using medical records, ADA criteria, or ICD codes. These inconsistencies likely contributed to the observed between-study variance. Finally, sample characteristics (e.g., age distribution, duration of diabetes) and study design (cross-sectional vs. longitudinal) may underlie the heterogeneity.

The high burden of mental illness in T2DM has important clinical implications. Approximately one in five adults with T2DM had comorbid depression in our pooled analysis, highlighting its clinical importance, and over half had cognitive impairment, suggesting that routine screening for mood and cognitive disorders should be an integral component of diabetes care. Depression in individuals with diabetes has been associated with poor medication adherence and worse glycemic control; for example, prior studies have shown that treating depression can lead to improvements in blood sugar management (10–12). Similarly, undetected cognitive deficits can impair self-care and increase the risk of complications. Given that diabetes affects hundreds of millions of people globally [with 537 million adults affected in 2021 (7)] and that mental disorders contribute substantially to disability worldwide (1), failing to identify and manage psychiatric comorbidities could exacerbate the overall disease burden. These findings underscore the need for integrated care models, where endocrinologists, primary care providers, and mental health professionals collaborate. In practice, this could mean incorporating depression questionnaires or cognitive screening tools into diabetic clinics and ensuring access to psychiatric evaluation and treatment for T2DM patients.

This review’s strengths include a comprehensive approach and a large overall sample. By pooling data from multiple countries and settings, we achieved greater statistical power than any single study. We assessed several psychiatric conditions (e.g., depression, MCI, and schizophrenia), providing a broad picture of mental health in T2DM. The review was conducted according to the PRISMA guidelines (23), with predefined criteria and independent data extraction, enhancing the transparency and rigor. However, there are some limitations that must be acknowledged. The included studies were highly heterogeneous in terms of methods and study populations, which limits the comparability of the results. Notably, only two studies addressed schizophrenia, resulting in an unreliable pooled estimate and extreme heterogeneity (25). Many studies were cross-sectional, precluding assessment of causality or temporal trends. Diagnostic criteria varied widely (e.g., self-report scales, clinical interviews, and registry codes), which may have introduced measurement bias. Furthermore, we could not quantitatively analyze some comorbidities (such as anxiety or bipolar disorder) due to the limited number of available studies. Publication bias is also a concern, although formal testing was limited by the small number of studies for certain outcomes.

Future research should address these gaps. Prospective cohort studies are needed to clarify the incidence and temporal relationship of psychiatric disorders in diabetes. Such studies could build on existing longitudinal data (e.g., national diabetes and mental health registries). Standardizing diagnostic methods would improve comparability, for instance, by using validated scales or clinical interviews across studies. Further research is also needed on understudied comorbidities. Our review highlights depression and MCI, but other conditions such as anxiety disorders, bipolar disorder, and substance use have been reported in individual patients and warrant systematic investigation. Finally, mechanistic studies—examining genetic, neuroendocrine, or lifestyle factors—could help explain why T2DM patients are vulnerable to mental illness. In summary, further research, especially large, multicenter, longitudinal studies, will be essential for understanding and ultimately mitigating the psychiatric burden in diabetes care.

## 5 Conclusion

This systematic review and meta-analysis demonstrates that psychiatric comorbidities, particularly depression and cognitive impairment, are highly prevalent among individuals with type 2 diabetes mellitus. The pooled prevalence of depression was 19%, while 39% of patients exhibited evidence of cognitive impairment, underscoring a critical need for routine psychiatric screening in diabetes care. In contrast, schizophrenia was rare but showed wide variation across studies, reflecting methodological and population differences. These findings highlight the bidirectional relationship between diabetes and mental health, emphasizing that integrated care approaches are essential to improve patient outcomes. Despite heterogeneity across studies and limited data for certain conditions, our analysis provides robust evidence that the psychiatric burden in T2DM is substantial and clinically significant. Future research should prioritize longitudinal, multicenter studies using standardized diagnostic methods to clarify causal pathways and to guide targeted interventions.

## Data Availability

The original contributions presented in the study are included in the article/supplementary material, further inquiries can be directed to the corresponding author.
